# The Pleiotropic Role of Retinoic Acid/Retinoic Acid Receptors Signaling: From Vitamin A Metabolism to Gene Rearrangements in Acute Promyelocytic Leukemia

**DOI:** 10.3390/ijms20122921

**Published:** 2019-06-14

**Authors:** Maria Rosa Conserva, Luisa Anelli, Antonella Zagaria, Giorgina Specchia, Francesco Albano

**Affiliations:** Department of Emergency and Organ Transplantation (D.E.T.O.), Hematology Section, University of Bari, 70124 Bari, Italy; mariarosaconserva@gmail.com (M.R.C.); luisa.anelli@uniba.it (L.A.); antonellazagaria@hotmail.com (A.Z.); giorgina.specchia@uniba.it (G.S.)

**Keywords:** Retinoic acid receptors signaling, chromosomal rearrangements, acute promyelocytic leukemia

## Abstract

The family of retinoic acid receptors (RARs: RARα, -β, and -γ) has remarkable pleiotropy characteristics, since the retinoic acid/RARs pathway is involved in numerous biological processes not only during embryonic development, but also in the postnatal phase and during adulthood. In this review, we trace the roles of RA/RARs signaling in the immune system (where this pathway has both an immunosuppressive role or is involved in the inflammatory response), in hematopoiesis (enhancing hematopoietic stem cell self-renewal, progenitor cells differentiation or maintaining the bone marrow microenvironment homeostasis), and in bone remodeling (where this pathway seems to have controversial effects on bone formation or osteoclast activation). Moreover, in this review is shown the involvement of *RAR* genes in multiple chromosomal rearrangements generating different fusion genes in hematological neoplasms, with a particular focus on acute promyelocytic leukemia and its variant subtypes. The effect of different RARs fusion proteins on leukemic transformation, on patients’ outcome, and on therapy response is also discussed.

## 1. Metabolism of Vitamin A

**Figure 1:**
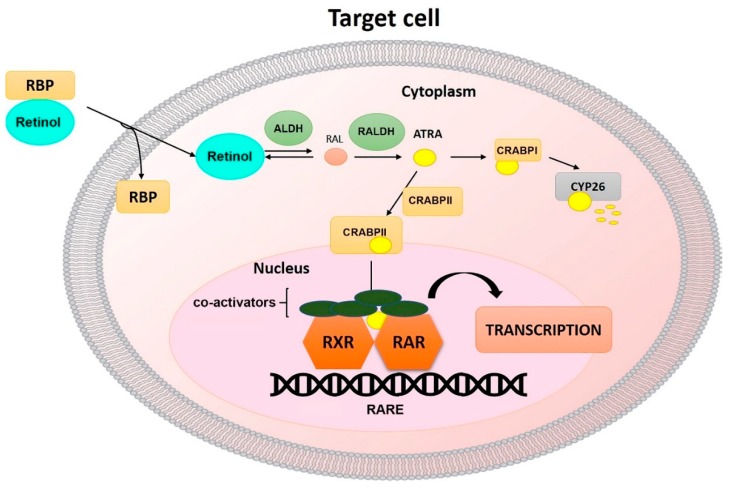
Mechanisms of RA signaling.

It is well noted that vertebrates, including humans, cannot synthesize vitamin A de novo but it should be introduced with the diet [1]. More than 70% of vitamin A is present as retinyl esters, that we find in foods like eggs, liver, bottled milk, or fortified cereals; or as carotenoids (e.g., β-carotene) in vegetables such as carrots or spinach. Vitamin A uptake is carried out by enterocytes with subsequent incorporation into chylomicrons. Eventually, these can be further processed by enterocytes or released into the circulatory system, where they are transported to target tissues. Although the dietary retinoids can meet different destinies, about 66–75% is taken up by the hepatocytes where it can be stored as retinyl esters or hydrolyzed to retinol [1,2]. Meanwhile, the remaining ones are taken up by extra-hepatic tissues such as white adipose tissue, skeletal muscle, heart, lungs, and kidneys [3]. Retinol, once released into the circulation, binds to the retinol-binding proteins (RBPs); these interacting with the RBP receptor, STRA6, mediate the retinol cellular uptake, so that it can be processed [4] (Figure 1). Once entered into a cell, retinol can endure reversible action by ubiquitous alcohol dehydrogenases (ADHs) to form the retinaldehyde (RAL) [5]. The cells that produce retinoic acid (RA) uniquely express the retinaldehyde dehydrogenases (RALDHs) that irreversibly converts the RAL into RA [6]. Newly synthesized RA binds to cellular RA-binding proteins (CRABPs). If bound to CRABPI, RA is transferred to CYP26 to be degraded, while if bound to CRABPII or FABP5, RA is relocated to the nucleus, where they interact, respectively, with nuclear retinoic acid receptors (RARs) or peroxisome proliferator-activated receptors (PPARs), promoting the transcriptional activity of RA target genes [7,8]. RA can also express its function outside the cell, showing a paracrine effect on nearby cells or remaining in circulation [5]. Several RA isoforms have been identified, the predominant one in most tissues is represented by all-trans RA (ATRA), responsible for most of the biological effects of vitamin A [5,9]. The importance of vitamin A, and therefore of its biologically active metabolites, has long been known, since it has a strongly pleiotropic role that shows from embryonic development. Not only that, it is important that a particular homeostasis is maintained, since both the deficiency and excessive presence of this vitamin can compromise embryonic development, causing, respectively, severe defects and showing teratogenic abilities [10,11,12,13,14,15]. It also follows that vitamin A levels are certainly critical even in the adult phase, as they influence the correct functionality of the immune, visual and reproductive system [16,17].

## 2. Retinoid Receptors

Retinoid receptors are part of the nuclear hormone receptor superfamily and comprise two subgroups which each contain three subtypes, the RARs (RARα, -β, and -γ) and the retinoid X receptors (RXRα, -β, and-γ). For each subtype, two or more isoforms can be identified, due to the alternative RNA splicing, resulting differently in the N-terminal portion [18,19]. RARs and RXRs are the first mediators of the effects of retinoids, both present a dimerization domain, a ligand-binding domain, and a DNA-binding domain with a zinc finger that binds to RA response elements (RAREs) in the promoter of their target genes [20,21,22,23,24,25]. In the presence of ATRA, RARs dimerize with RXRs, forming a heterodimer that acts as a transcription factor, activating the RAREs regions in the promoter of the target genes [24,26]. In particular, the presence of the ligand induces a conformational change in the ligand-binding domain of the receptor, facilitating the binding and recruitment of coactivators such as histone acetyl transferases (HATs) including nuclear receptor coactivator 3 (NCoA3 or ACTR). In the absence of ATRA, the RAR/RXR heterodimer binds to RAREs and represses the transcription through the recruitment of corepressors such as the histone deacetylases (HDACs) or nuclear receptor corepressor (NCoR) [24,25,27,28]. Each subtype of RAR presents a different sensitivity at different concentrations of ATRA: RARα activation occurs in the presence of high concentrations of ATRA, in contrast with RARγ activation which requires the lowest amount of ligand [29]. As already mentioned above, ATRA could bind to other nuclear receptors like PPARs (α, β/δ, γ). The FABP5-ATRA complex, translocating in the nucleus, interacts with the PPARs, which in turn, dimerize with RXRs, activating the target genes’ transcription. Considering that the binding affinity of CRABPII-RARs for ATRA is much stronger than that of the FABP5-PPARs complex, it follows that the transcriptional activity mediated by RARs is dominant [30]. In addition to RARs, RXR and PPARs, retinoids can bind retinoid-related orphan receptors (ROR) β and γ [31,32]. RORs do not form heterodimers with RXR but regulate gene transcription by binding as monomers to specific ROR response elements (ROREs) in target genes [33,34]. The actions of retinoids can be subdivided into genomic actions, as they interact with different nuclear receptors that regulate *RAREs*, peroxisome proliferator response elements (*PPREs*) and *ROREs* in target genes, and rapid non-genomic/non-classical actions [25,35]. Indeed, retinoid receptors reside in the nucleus, but in particular conditions and in some cells they move into the cytoplasm, where they can regulate the translation and act as monomers or complexes with various cellular factors, in the cascades of the kinases by participating in signaling events [25]. For instance, in neuronal cells, in the absence of a ligand, RARα can be exported to the cytoplasm and behaves as a RNA-binding protein that associates to mRNAs in a sequence-specific manner and inhibits their translation [36,37,38], such as glutamate receptor 1 (GluR1)-encoding mRNA. While, in the presence of a ligand, RA interaction releases RARα from the mRNA, promoting translation and resulting in protein expression [38]. Among these non-classical retinoid actions, it was also seen that ATRA can induce a rapid phosphorylation of cyclic AMP response element-binding protein (CREB), which translocate to the nucleus to activate gene target transcription [25,35]. This effect is not limited to ATRA but can be exerted by retinol [39]. Therefore, these non-canonical retinoid actions connect extranuclear sensing with genomic activation. Furthermore, these additional extranuclear skills enhance the complexity of the functions of retinoids and, therefore, their pleiotropic effects [8].

## 3. RA and RAR Signaling during Embryonic Development

The signaling path of retinoids plays an essential role from embryonic development in all superior animal species ranging from fish to humans [40]. Through the use of animal models, it has been shown that the presence of ATRA is necessary for the development of different organs and tissues, including the eye, hind-brain, spinal cord, heart, lung, pancreas, and skeleton [41]. During the development, ATRA influences the cellular commitment, in particular, some cells differentiate and form the belonging tissue in the presence of high concentrations, while others require low concentrations [42]. Studies in mice have shown that during embryonic development, three distinct waves of development of hematopoiesis occur at different times and in well-defined sites. The first primitive wave, which is seen in the yolk sac, generates, predominantly, erythroid cells that express only embryonic globins, as well as some macrophages and megakaryocytes. Only for these cells, terminal differentiation also begins in the yolk sac. The second wave is called the “wave of erythromyeloid progenitors”, in which these progenitors leave the yolk sac and begin, at the level of the fetal liver, the fetal hematopoiesis with the production of erythroid cells which express the final adult globes. The third wave, however, occurs much later in the large arteries of the embryo and probably also in the extra adjacent embryonic tissues in the yolk sac and in the placenta and generates true self-renewing, multipotent hematopoietic stem cells (HSCs) [43]. Furthermore, it has been reported that when the HSCs emerge, both *RARΑ* and *RARG* are expressed. Interesting were the results that Chanda et al. obtained in vitro, treating immature hemogenic endothelial cells (ECs) precursors with a Rarα agonist, AM580, that has greatly improved the maturation of transplantable HSCs, while the use of a Rarγ agonist showed no beneficial effect and the use of ATRA showed a mild effect. Not only their studies suggested that during the transition of hemogenic endothelium to HSCs, the effect of RA signaling occurs concurrently with the downregulation of Wnt signaling [44]. Altogether, these studies implied that RA signaling is necessary for normal HSC development and is sufficient to induce maturation of ECs to functional HSCs in culture. Furthermore, this signaling influences the development and differentiation of primary lymphoid organs [45]. RA and RARs play an important regulatory role in the maintenance of thymic epithelial cells homeostasis, and the thymic mesenchymal cells are the major RA resource during embryonic development [46]. In addition, RA signaling plays an important role in the development of secondary lymphoid organs (SLOs). Indeed, mice that developed under vitamin A deficient conditions showed much smaller and fewer SLOs than control mice [47].

## 4. The Pleiotropic Roles of RA/RAR Signaling: From the Immune System to the Bone Remodeling

RA and RARs play an important role in the controlling of postnatal immune functions, especially at the mucosal border of the intestine. This signaling plays an important role especially in the maintaining of the balance between an optimal protective immunity and effective peripheral tolerance. However, functions of RARs signaling are highly contrasting, in fact RA is frequently associated with immunosuppressive roles, but at the same time RA and RARs can function as initiators of inflammatory response and protective immunity [8]. In addition, ATRA has been shown to have pleiotropic effects on hematopoietic cells, enhancing HSC self-renewal while also increasing differentiation of more mature progenitors [48]. Anyhow, the effects of such signaling are really manifold, for example hypervitaminosis A promotes, also, skeleton fragility by increasing osteoclast formation and decreasing cortical bone mass [9].

Retinoids influence the maturation and antigen-presentation function of dendritic cells and their ability to trigger T cells both in a tolerogenic and inflammatory manner [8]. In a mouse model of autoimmune experimental encephalitis, RA counteracts inflammation and downregulates maturation markers on DCs, thus impairing their antigen-presenting capacity [49]. At the same time, in the presence of infections, RA promotes the pro-inflammatory action of dendritic cells and T cell activation. RA also can act in a contrasting way on macrophages; for example, in the presence of LPS, it improves IL-10 production, which in turn feeds back on macrophages, further limiting the production of pro-inflammatory cytokines [50]. Alternatively, in the presence of granulocyte-macrophage colony-stimulating factor (GM-CSF), RA promotes the phagocytic antigen presentation function of macrophages leading to induced regulatory T cell (Treg) formation and immune tolerance [51].

The effects of RA signaling on T cells depend on several variables, including the intracellular dose of RA, the RAR isoform that is expressed, the specific cytokine environment, and the nature of the responding T cell. T cells do not produce RA, but they express constitutively *RARA* and *RARG* and induce *RARB* in certain conditions. However, most of the effects of retinoids in T cells are mediated by the RARα subtype [52]. Treg cells operate through different mechanisms, but it appears that the expression of the forkhead-box-P3 transcription factor (FoxP3) is critical for their function. In particular, there is a cell population that is called induced Tregs (iTreg), which is characterized by a high expression of FoxP3. RA promotes transforming growth factor-β (TGF-β)-dependent Foxp3-expressing Treg differentiation, but, at the same time, suppresses Th17 cell differentiation [53]. Furthermore, RAR antagonists inhibit iTreg generation, whereas the addition of RA to cocultures with splenic DCs drastically increases TGF-β-driven Treg differentiation in vitro [53]. iTregs generated in vitro, in the presence of RA, were able to control inflammation when transferred in mouse models of arthritis [54] and type 1 diabetes [55]. Nevertheless, the differentiation of the Treg generated in the thymus (tTreg) in response to self-antigens not dependent on RA signalling, in fact is intact in *Rara*−/− mice [52]. However, RA signaling alone is not sufficient to induce Foxp3 expression, but it is the synergy with the TGF-β, which in turn increases the expression of RARα [56,57] that allows the generation of iTregs [53,58,59]. As is already known, the effects on T cells are closely related to the type of T cell that is considered. In fact, while in the naive T cells, the RARα signal fosters the Th2 polarization on Th1 [60,61,62]; in already polarized Th cells, RARα promotes a stable expression of the Th1 phenotype, to the detriment of Th17, that regulates enhancer activity of Th1-signature genes while repressing genes that regulate Th17 cell fate [63]. These contrasting effects of RA/RARα signaling are mainly indirect, as they regulate the Th1/Th2 balance by regulating the cytokines production by antigen-presenting cells (APCs) [64,65,66].

The effects of RA signaling on B cells are numerous in this case, but the main influence regards the immunoglobulin (Ig) class switching, controlled by the pathway both in a direct and indirect manner. The RA/RAR system is, in fact, involved both in the germinal transcription of the Ig gene and in the expression of the coreceptor molecules necessary for B cells activation, both in the expression of the activation-induced cytidine deaminase and in the expression of cell surface molecules typical of mature, immature or plasma cells and in the formation of germinal centers, where class switching and affinity maturation of antibody occurs. RA, cooperating with other factors, is necessary to promote an antibody response against T-cell-dependent antigens such as tetanus toxoid and against T-cell independent type 2 antigens such as pneumococcal polysaccharides [67]. Through increased activity of mitogen-activated protein kinase (MAPK) and nuclear factor kappa-light-chain-enhancer of activated B cells (NF-kB) signaling, RA promotes the expansion and production of antibodies by memory B cells [68]. In contrast, RA inhibits the proliferation of B cells, arresting them in the G0/G1 phase of the cell cycle to allow their differentiation in plasma cells [69,70]. RA acts by decreasing the expression of Pax-5, which with the repressor Bcl-6, represses the differentiation of plasma cells and increases the expression of the transcription factor Blimp-1 that cooperates with XBP-1 to direct the class switching and Ig secretion [71]. RA signaling influences the switching of the IgG class, but is much more significant for IgA-producing cells. For example, mice with vitamin A deficiency conditions show a marked decrease in IgA-secreting plasma cell accumulation and reduced IgA production in the intestine, which leads to increased susceptibility to local infections [72,73,74]. At the same time, however, serum IgA levels remain normal, indicating that it is probably not absolutely necessary for switching and production of the IgA class [74,75]. Furthermore, RA has been shown to inhibit the switching of the IgG1 class both in vitro and in vivo, while it shows no effect on the IgM class [76]. In addition, in some in vitro conditions, it inhibited the IgE production, but this has not been observed in vivo [77]. As in other immune cells, the different effects of the RA signal in B cells depend on several factors such as the type of antigen from which they are stimulated, the stage of differentiation of the responding cell, and the differential expression of *RAR* isoforms.

RA/RAR signaling also influences the behavior of various innate lymphocytes, such as innate lymphoid cells (ILCs) [78,79], TCRγδs [80], NKT, and NK cells. In fact, the presence of RA promotes the gene expression of the RA-inducible genes such as *RAE-1* and *MICA/B*, which activate NKT cells and NK cells, respectively [81]. It follows that even in this case there are two conflicting effects. The number of NKT and NK cells is positively regulated by the levels of retinol available, but at the same time the RA effects on these cells is predominantly suppressive, as seen, for instance, in NKT cells that when activated by the presence of RA, reduce the production of INFγ [82]. So even in this case, the RA/RAR system can have both a promoting and inhibitory effect, interacting positively or negatively with other signaling cascades and pathways [8].

Several studies reported that hypervitaminosis A causes both decreased bone mass and enhanced osteoclast formation [83,84]. Kneissel et al. [83] and Lind et al. [84] both performed a treatment of male or female rats, respectively, with Ro 13-6298, a third generation retinoid, for 4 days and with a mixture of retinyl palmitate/retinyl acetate for 7 days, obtaining a decrease of cortical bone mass and an increase of osteoclasts at the periosteal side of cortical bone. However, Kneissel et al. identified a decrease in the number of trabecular osteoclasts without any effect on trabecular bone mass; on the contrary, Lind et al. reported a decrease in the trabecular bone mass but with an unaltered number of osteoclasts. Since the decrease in bone mass is not always associated with an increase in the number of osteoclasts, it was speculated that other cells may be responsible for the action of vitamin A. Raisz et al., through the use of organ culture, showed that vitamin A increases bone resorption, accompanied by an increase in the number of osteoclasts. However, this number is significantly lower than that induced by the parathyroid hormone (PTH), probably because the bone resorption caused by vitamin A is given, firstly, by an increase in the release of lysosome enzymes, rather than by a rising number of osteoclasts [85]. The evidence that retinoids stimulate bone resorption and osteoclast formation was given by Conaway et al., which demonstrated that ATRA increases mRNA and the receptor activator of nuclear factor kappa-β ligand (RANKL) protein expression, and at the same time transiently interacts with osteoprotegerin (OPG) mRNA, decreasing protein expression. In fact, this effect was inhibited by exogenously added OPG. Nonetheless, it has not been identified that the type of cell in the presence of ATRA increases the *RANKL* expression. But not only that, from this study it has emerged that who mediates the effects of ATRA on RANKL and on bone resorption is RARα [86]. Other studies have used bone marrow cultures containing bone marrow stromal cells and hematopoietic cells, including osteoclast progenitors. In this case, the effect of vitamin A has been controversial, since ATRA has no stimulating effect on the formation of osteoclasts [87], nor does it induce the expression of osteoclastogenic genes. Probably, because such cells present in the bone marrow respond differently to retinoids, compared to the different bone cells used in previous studies. Unexpectedly, ATRA inhibits osteoclastogenic growth even in the presence of 1,25 (OH) 2-vitamin D3 in culture of rat bone marrow [88] and in co-cultures containing mouse bone marrow cells and mouse calvarial osteoblasts [89]. These results suggest that ATRA, rather than acting in a later phase of osteoclastogenesis, acts by inhibiting the osteoclastic progenitors differentiation. Indeed, Conaway et al., have shown that ATRA inhibits signals downstream of the activated nuclear factor receptor kappa β (RANK), giving an explanation of osteoclastogenic progenitor’s differentiation inhibition. Furthermore, the use of RARβ/γ (GR103) and RARγ (A7980) agonists in bone marrow macrophages cultures has shown that the inhibitory effect of osteoclastogenesis is less powerful, suggesting that RARα is the most important RAR-mediating retinoid-induced inhibitor of osteoclastogenesis [86]. The activity of retinoic acid receptors seems to be controversial even in bone formation. Several studies have stated that vitamin A has stimulatory effects on bone formation [84]. RA/RAR signaling, in this case, also shows often controversial effects, since the possibility exists that it is the ratio between vitamin A and other factors (such as vitamin D) which is important for bone mass rather than vitamin A itself.

## 5. RA/RAR Signaling Involvement in Apoptosis

The most recognized functions of retinoids are cellular proliferation and differentiation, but an involvement of RA in proapoptotic activities has been reported. Retinoids can induce the death of some cell types [90] and inhibit apoptosis in others [91]. Indeed, among the *RAR* target genes are identified caspases, Bcl-2 proteins, transcription factors that regulate apoptosis, and genes involved in DNA fragmentation. In particular, it has been reported that in MCF-7 mammary carcinoma cells, at the level of the second intron of the gene encoding for caspase 9, a *RARE* that mediates the ability of RAR to modulate the expression of caspase 9 is present, thus activating the intrinsic apoptotic pathway. In addition, it seems that the expression of the CRABPII-binding protein considerably enhances the ability of RAR to induce apoptosis through the upregulation of the expression of caspase 9 [92]. Mrass et al., reported that, also in keratinocytes, RA upregulates the expression of caspase 3, 6, 7, and 9 [93]. RA, also, can induce apoptosis, modulating the expression of both proapoptotic and antiapoptotic Bcl-2 proteins; indeed, in neuroblastoma cells [94], metastatic melanoma [95] and myeloblastic leukemia cells [96], it has been confirmed that apoptosis induced by RA is accompanied by downregulation of Bcl2 with subsequent activation of caspases 9 and 3. Furthermore, several reports suggested that RA can upregulate the expression of the tumor suppressor p53 in pancreatic cancer cells [97], thymocytes [98], telomerase-immortalized Barrett’s cell lines [99], metastatic melanoma cells [95], and myeloblastic leukemia cells [96]. Moreover, it has been seen that RA may be involved in extrinsic apoptotic signaling in postmaturation NB4 APL cells through the induction of expression of interferon regulatory factor-1 (IRF-1), which in turn upregulates the death ligand TRAIL [100,101]; in human lung cancer and in leukemia cells, RA can stimulate the expression of the receptor for the death ligand TNFα [102,103]. In addition, RA could be involved in activating the extrinsic pathway mediated by the Fas death receptor, although the underlying mechanism is not completely understood [104]. Notably, RARβ_2_ isoform is considered to be a tumor suppressor, as it mediates the carcinoma cell growth inhibition activity exerted by RA. In particular, it has been reported that in many malignant tissues, epigenetic silencing phenomena occur at the level of the *RARB* promoter [105,106,107,108], leading to the loss of the RARβ_2_ isoform [109,110,111]; indeed, the methylation status of *RARB* promoter has been used as a biomarker for malignancy or to monitor the efficiency of anticancer drugs in clinical trials [112,113]. Nevertheless, in some cellular contexts, RA preserves cell survival; in fact, several reports suggested thatit is critical for neuronal survival [114,115,116,117], inhibits radiation-induced apoptosis in keratinocytes [118], prevents retinal progenitor cells [119] and cardyomyocite apoptosis [120], and counteracts TNFα-induced apoptosis of lung epithelial cells [121]. In addition, RA seems to exert antiapoptotic activities even in the MMTV-*neu* mouse model of breast cancer [30,122]. The double role that RA can assume suggests that probably the increase of cells proliferation is not mediated by RARs, whose target genes are usually involved in inhibition of cell growth. Indeed, some studies have shown that some antiapoptotic activities of RA are mediated by the alternative RA receptor PPARβ/δ [30], whose target genes are involved in proliferative and antiapoptotic activities [123,124]. So the ability of RA to favor apoptosis rather than cellular survival, or conversely, is closely related to the expression levels of the two RA binding proteins, since CRABPII shuttles RA towards RAR and FABP5 versus PPARβ/δ [30]. Accordingly, it has been reported that the malignant progression in human cancers is associated with malignant progression [125]. Consequently, in some tumors, the expression of PPARβ/δ target genes is favored rather than RAR, thus favoring tumor growth [126].

## 6. The Complex Role of RARs in the Regulation of Hematopoietic Stem Cells (HSCs)

**Figure 2:**
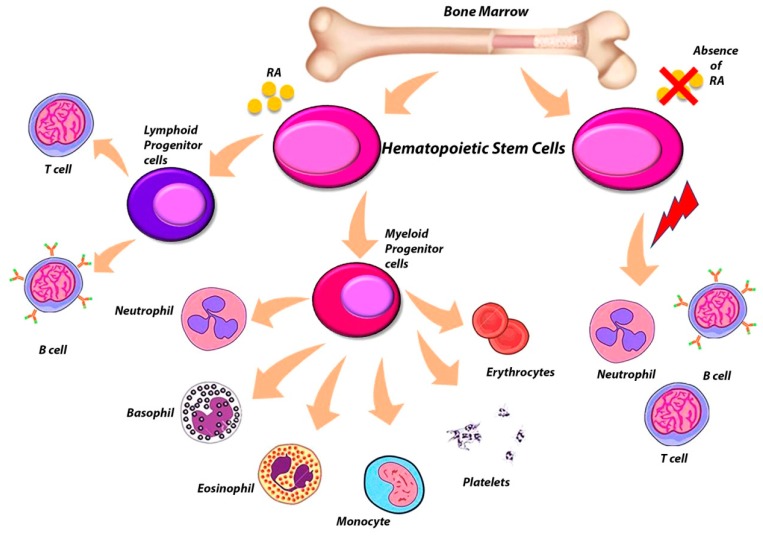
Effect of RA absence on HSCs differentiation.

It has long been known that RARs are nuclear receptors that play an important role in the proliferation and differentiation of numerous cell lines. Several studies have shown that RARs have a direct role both in hematopoiesis and in non-hematopoietic cells present in the bone marrow (BM) microenvironment and which also influence the correct process of hematopoiesis [127] (Figure 2). The utilization of ATRA to differentiate leukemic cells block at the promyelocytic stage in patients with APL is a first example of the importance of retinoids signaling in the process of differentiation of hematopoietic cells [128]. The different subtypes of RAR are expressed differently in hematopoietic cells, in fact several studies have used RAR-knockout mice to better establish the roles of the individual RAR subtypes in hematopoiesis (Table 1). The RARα and RARγ receptor subtypes are expressed a lot both in stem cells and hematopoietic progenitors, as well as in more mature hematopoietic cells. Only *Rarb2* is expressed by hematopoietic cells, as it is the only isoform presenting a RARE in its promoter region [129]. Not only that, *Rarb* has been identified as the most important RAR for the ATRA-induced HSC self-renewal, since after being treated with ATRA, this isoform is much more expressed in HSCs, compared to *Rara* and *Rarg* [130]. *Rarb*^−/−^ mice, which are null for all four *Rarb* isoforms, developed normally and retained the capacity to respond to ATRA [131]. On the contrary, *Rara*^−/−^ and *Rarg*^−/−^ mice exhibited defects in several tissues and, initially, showed a high post-natal mortality [132,133]. Although these mice were housed in a clean environment, their condition improved, especially for *Rara*^−/−^ mice [129]. While *Rarg*^−/−^ mice continued to show a certain postnatal lethality even living in clean housing, only some reached 12 months of age [129]. *Rarg*^−/−^ mice showed a decrease in bone mass and a significant increase in granulocytes in the peripheral blood (PB), BM and spleen, thus developing a myeloproliferative-like syndrome [134], and exhibited defects both in erythropoiesis and in B lymphopoiesis [134,135]. Nevertheless, Kastner et al. reported that mice null for *Rara* isoforms have a granulocyte population that appears normal in vivo, indicating that probably RARα is not indispensable for granulocyte maturation in vivo [136]. Not only that, *Rara*^−/−^ mice also exhibited normal HSC numbers and function, demonstrating that RARα is dispensable for HSC maintenance (98). Subsequent studies have, also, shown that male *Rarg*^−/−^ mice show an increase in osteoclastogenesis with a consequent loss of trabecular bone mass [137]. Through transplantation studies, it has been seen that the expression of *RARG* is fundamental in the BM microenvironment so that a correct hematopoiesis occurs [138]. Therefore, the abnormalities reported by *Rarg*^−/−^ mice are not due to an intrinsic cellular defect but were instead due to an aberrant *Rarg*^−/−^ BM microenvironment [135]. Mice in which *Rarg* was deleted in neural crest-derived mesenchymal stem cells targeted by *Nestin*-*Cre* and their progeny exhibited impaired BM B lymphopoiesis and thymic T lymphopoiesis; however, the mice maintained normal number and functionality of HSCs [139] . Therefore, mice conditionally deleted *Rarg* in more primitive limb bud-derived mesenchymal stem cells (MSCs), and their progeny using *Prrx1-Cre* showed changes in trabecular bone and longitudinal bone growth, as for *Rarg*^−/−^ mice. However, the two phenotypes are not exactly identical, as these *Prrx1:Rarg^Δ^^/^^Δ^* mice also show altered angiogenesis and B lymphopoiesis, showing how the activity of RARγ is a critical regulatory key for the presence of a healthy BM microenvironment [127]. In particular, this study highlights substantial differences between male and female *Prrx1:Rarg^Δ^^/^^Δ^* mice. Indeed, the difference in the trabecular bone mass was more pronounced in males than females; this is concordant with the presence of more trabecular osteoclasts in males, whereas *Prrx1:Rarg^Δ^^/^^Δ^* females exhibit more trabecular osteoblasts. These results suggest that the cellular mechanisms by which RARγ regulates trabecular bone mass are either different in males and females or predominate to different extents in males and females. In addition, *Prrx1:Rarg^Δ^^/^^Δ^* male mice demonstrated altered BM-B lymphopoiesis, presenting an increase in the number of B lymphocyte precursors in the BM, with elevated pro-B and pre-B lymphocytes, but no changes in the more primitive pre-pro-B lymphocytes. However, this expansion of B lymphocyte precursors is not reflected in the mature B lymphocyte population, as the number of splenic and mature PB B lymphocytes was unchanged. On the contrary, *Prrx1:Rarg^Δ^^/^^Δ^* females not exhibiting a significant increase in BM leukocytes presented an increase in BM granulocytes, but no other significant differences were detected in the other hematopoietic cell populations [127]. It is possible to suppose that in male mice the B lymphopoiesis is related to the high presence of osteoclasts, since the administration of the anti-resorptive zoledronic acid (ZA) led to impaired B lymphopoiesis from the pre-pro-B lymphocyte stage, with no direct effect of ZA observed on the B lymphocyte cells [139]. This suggests that the altered osteoclastic function somehow affects normal B lymphopoiesis. In addition, *Prrx1:Rarg^Δ^^/^^Δ^* mice presented an altered BM vascularization, since they presented an altered *Vegf-a* expression that is the key regulator in the process of angiogenesis in BM [127]. In the BM, *Vegf-a* is expressed by osteoblast-lineage cells; hypertrophic chondrocytes; and some proliferating chondrocytes [140,141]; and some hematopoietic cells, including myeloid progenitors and megakaryocytes [142]. Green et al., revealed that Vegf-a was upregulated in the megakaryocytes in the bone marrow of the *Prrx1:Rarg^Δ^^/^^Δ^* mice [127]. Since Vegf-a enhances osteoclastic differentiation, survival and resorptive activity [143], it is possible to hypothesize that in *Prrx1:Rarg^Δ^^/^^Δ^* males the increase of *Vegfa* contributed to the elevated trabecular osteoclasts and lower trabecular bone mass through the stimulation of osteoclast differentiation and recruitment. However, the enhanced vascularization in *Prrx1:Rarg^Δ^^/^^Δ^* females may have increased the osteoblast recruitment to the bone surface, contributing to the increased numbers of osteoblasts observed in these female mice [127]. These results confirm the key role of RARγ in limb bud-derived MSCs in the BM microenvironment as a regulating factor of endochondral bone formation, angiogenesis, osteoclastogenesis and B lymphopoiesis [127]. Several studies with knock-out mice have demonstrated that RARα and RARγ appear to have opposing effects in adult hematopoietic cells. Indeed, RARα has been shown to enhance myeloid commitment and granulocytic differentiation, explaining the block on terminal granulocytic maturation observed in promyelocytic APL leukemic blasts [144]. In contrast, RARγ is important in maintaining homeostasis in the various processes that occur in the BM microenvironment.

## 7. Alternative *RARs*-Rearrangements Resemble Acute Promyelocitic Leukemia (APL)

**Figure 3:**
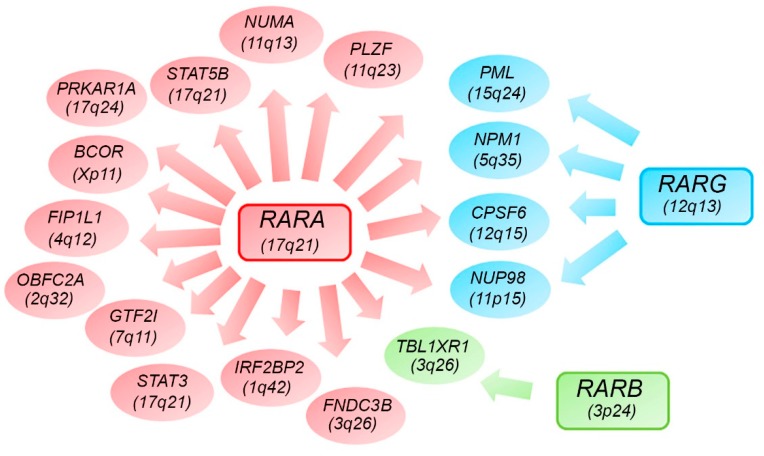
Diagram showing possible fusion genes involving the *RAR* family gene members (*RARA*, *RARB* and *RARG*) with some partner genes being shared.

The fusion genes generated by chromosomal translocations play a critical role in the regulation of cell proliferation, differentiation and apoptosis in hematological malignancies [145]. APL is a unique disease entity in AML, characterized by a large proportion of patient carriers t(15;17)(q24;q21) involving the *promyelocytic leukemia* (*PML*) gene at chromosome band 15q24 and the *retinoic acid receptor alpha* (*RARA*) gene at 17q21, generating an aberrant *PML-RARA* fusion gene [140,146]. Anyway, typical APL is characterized by recurrent PML-RARα expression, and it is sensitive to ATRA and arsenic trioxide treatment [147,148,149,150]. However, there is a subset of patients with APL t(15;17)(q24;q21), in which *PML-RARA* fusion cannot be detected [147]. Currently, several *RARA, RARB, or RARG* fusions have been reported with at least 17 alternative partner genes in patients with APL, including *PLZF, NPM1, NUMA, STAT5B, PRKAR1A, BCOR, FIP1L1, OBFC2A, GTF2I, TBLR1, IRF2BP2, NUP98, FNDC3B, PML, STAT3, CPSF6*, and so forth [140] (Figure 3). Recently, different *RARG*-rearrangements have been identified, including *NUP98-RARG*, *PML-RARG* and *CPSF6-RARG*. The first AML patient with *RARG*-rearrangement, *NUP98-RARG*, was reported in 2011 [146]. The nucleoporin 98 gene (*NUP98*) located at chromosome 11p15 is recurrently involved in a variety of rearrangements in both myeloid and lymphoid malignancies [151,152]. NUP98 is a structural component of the nuclear pore complex (NPCs) responsible for protein and RNA transport [153]. NUP98 contains a N-terminal domain with a GLFG repeat (for Gly-Leu-Phe-Gly) that has been shown to activate transcription, providing docking sites for nuclear transport to conduct RNA and protein traffic between the nucleus and cytoplasm. Chimeric transcripts formed by the *NUP98* N-terminal GLFG repeats fused to the C-terminus of the partner proteins are expressed in all *NUP98* fusions reported, suggesting that the *NUP98* N-terminus may be important for leukemogenesis [154,155]. Several studies [146,156], reported a case of acute myeloid leukemia with a clonal translocation t(11;12)(p15;q13) displaying morphologic and immunophenotypic features resembling the classical hypergranular subtype of APL. Through the sequence analysis, they identified the presence of a fusion transcript in which the *NUP98* exon 12 was fused in-frame to *RARG* exon 4, whereas the reciprocal fusion transcript was not identified. Since the fusion was in-frame, the open reading frames of both genes in the fusion transcript were retained. The *NUP98* 5′-region encoding the GLFG-repeat and the GLEBS-like motifs were fused to the 3′-region of *RARG*, which included the DNA- and ligand-binding domains of the gene. Qiu et al. reported that the chimeric protein thanks to the characteristics preserved by the two fused portions of the two proteins, acquires new characteristics and capabilities. The RARG DNA-binding domain favors a new cellular localization of the protein, while the portion of NUP98 confers aberrant homodimerization properties, suggesting that both the two fused portions are required for the leukemogenic transformation. Furthermore, the transcriptional properties are similar to the RARA fusions identified in APL patients [157]. Despite the fact that clinical feature of *RARG*-rearranged leukemia is similar to APL, its treatment is totally different. Indeed, the patients described by Such and Zhang respond intermittently to treatment with ATRA, probably due to the absence of a *PML/RARA* rearrangement; so complete remission was achieved only by switching the therapy to chemotherapy a standard 3+7 regimen [146,156]. Such at al. also tested in-vitro sensitivity to ATRA of the patient leukemic blasts. The in-vitro study showed that AML with a *NUP98/RARG* rearrangement is resistant to ATRA [158] and is probably the NUP98 counterpart in the chimeric protein that mainly confers the refractoriness to ATRA treatment. On the contrary, Qiu et al. demonstrated that ATRA and RXR agonists can suppress the transformation mediated by the NUP98-RARG fusion [157]. Other different cases of atypical APLs allowed to identify another recurrent fusion gene as *CPSF6-RARG*. Cleavage and polyadenylation-specific factor 6 (CPSF6) is a large subunit of cleavage factor I, which is an RNA-binding protein complex that was originally identified as a central player in the alternative cleavage and polyadenylation process [159]. Several studies [159,160] reported different cases in which also *CPSF6-RARG* fusion transcript showed morphological and immunophenotypical features of classical APL. The patient described by Qin et al. [159] featured two types of *CPSF6-RARG* fusion transcripts. Both fusions were in frame, the major fusion was the fusion of *CPSF6* exon 4 with *RARG* exon 2, and the minor fusion had a 3-bp deletion at the 5′ end of *RARG* exon 2 compared with the major one. However, Liu et al. [160] reported two patients that both presented the breakpoint in 12q15 that were located at the intron 4 of *CPSF6* in both patients, while there are two breakpoints in the intron 3 or 5′ untranslated region and telomeric of exon 9 of *RARG*. So the 3′ region of *RARG* (from exon 1 or exon 4 to exon 9) was reversed and fused in frame with the 5′ region of the *CPSF6* gene (from exon 1 to exon 4), thus generating a longer and shorter transcript, both in frame. Both fusion proteins show an intranuclear diffusion and a transcriptional activity comparable with that of RARA or RARG. Miller et al. [161] reported instead *RARG-CPSF6* fusion in a patient with AML that appeared to have typical APL by standard histopathology and immunophenotyping. The patient showed a highly rearranged region (including multiple deletions, inversions, and intrachromosomal translocations) on chromosome 12 with break points in the *EIF4B*, *RARG*, and *CPSF6* genes. The rearrangement resulted in a fusion of *RARG* (in intron 9) to *EIF4B* (in intron 8) and a deletion after intron 8 of *EIF4B* that then fused into the intron leading into exon 6 of *CPSF6*. This complex rearrangement has generated a novel *RARG-CPSF6* in-frame fusion transcript. In this case, the expressed fusion protein has not been detected. Also, in all the cases just described, the treatment with ATRA did not allow the achievement of complete remission [158,159,160,161]. Ha et al. reported a novel *PML-RARG* fusion in a patient with apparent APL, which harbored a clonal translocation t(12;15)(q13;q22). They found two kinds of *PML-RARG* transcripts: The longer transcript was revealed as the fusion of *PML* exon 3 and the middle point of exon 1 of *RARG* while the shorter one was the fusion of *PML* exon 3 and exon 2 of *RARG.* By comparing the identified fusion sites with those of the classic *PML-RARA* fusion, it emerged that the fusion site of *PML* was the same as that of the bcr3 type of *PML-RARA,* but the larger part of *RARG* was fused compared to *RARA.* Also, in this case, a favorable outcome was achieved with standard chemotherapy [162]. It has also been reported that an AML case with morphology resembling APL characterized by *EZH2* gene mutation is associated with dysregulated *RARA* and *RARG* genes expression [163]. Osumi et al. performed a high-throughput sequencing analysis on APL cases without *RARA* translocation, finding a novel recurrent fusion of retinoic acid receptor-β (*RARB*). The fusion involved *RARB* that is located at 3p24 and *TBL1XR1* at 3q26, given by t(3;3) or an inv(3). This study demonstrated that TBL1XR1-RARB was an oncogenic protein having effects similar to those of PML-RARA; it homodimerized, diminished transcriptional activity for the retinoic acid pathway with a dominant negative effect on both *RARA* and *RARB*, blocked cell differentiation, and induced proliferation [164]. Interestingly, the deletion and mutations in *TBL1XR1* have been reported in various malignancies [165], and a previous study showed that TBL1XR1 was a component of the protein complex regulating the retinoic acid pathway [166]. However, in this case, the response of these patients with *RARB* translocation to retinoids was partial and was in line with the clinical finding of the resistance of *RARA*-negative APL to ATRA [164]. Lijun wen et al. also identified 19 patients with alternative *RARA or RARG* fusions, including *PLZF-RARA* fusions in 10 patients, *STAT5B-RARA* in 4, *STAT3-RARA* in 2, *CPSF6-RARG* in 2, and *TBLR1-RARA* in 1 patient. They analyzed the prognostic impact of APL with alternative *RARA* or *RARG* fusions, showing that the 3-year overall survival (OS) and leukemia-free survival (LFS) of APL with alternative *RARA or RARG* were worse than that of *PML-RARA* cohort. Indeed, among the 19 resembling APL patients involved in their study, 15.79% (3/19) were insensitive and 63.16% (12/19) were resistant to ATRA treatment. Also, NGS performed on APL patients with alternative *RARA* or *RARG* fusions revealed more mutations of *KMT2C*, *K-RAS*, and *GATA2*, but fewer mutations of *FLT3-ITD* when compared to APL patients with *PML-RARA* fusion [167].

## 8. Conclusions

The RA/RARs signalling is a complex pathway involved in several cellular processes whose effects are not always predictable, as they depend on the expression of the various RARs isoforms, on the amount of RA, and on the nature of the responding cell. Nevertheless, they regulate the cells of the immune system, bone remodeling and participate actively and not in the hematopoiesis processes. With regards to the role in immune system regulation, RA/RARs signaling functions are highly contrasting as this pathway is frequently associated with immunosuppressive roles or can also function as an initiator of the inflammatory response. A controversial role is also played in relation to bone remodeling as the RA/RARs signaling can stimulate or inhibit bone formation. Moreover, with regards to the effect on hemopoiesis, this pathway plays an important regulatory role in both myeloid and lymphoid cells maturation. It is now well known that *RAR* genes are often the protagonists of chromosomal alterations, mostly translocations, which involve the formation of several possible fusion genes frequently detected in patients with typical or atypical APL. Pathological phenotyping seems to overlap, but patients with atypical APL seem to have a poorer prognosis, due to resistance to ATRA. This is the reason why these cases with morphological features resembling APL are more reasonably classified as a subclass of AML. Thus, further investigation is needed to better understand the biology of *RARG* and *RARB* involvement in leukemogenesis, and the contrasting effects of the RA/RARs signalling in multiple cellular processes.

## Figures and Tables

**Table 1:** Summary of the phenotypic effects of knockout mice for RAR genes.

	Hematopoietic Cell Population	Bone Marrow Phenotype	Bone Marrow Defects	Reference
***Rara^−/−^* mice**	normal granulocyte population normal HSCs numbers and function	normal development	manifold defects in several tissues high post-natal mortality	[97,132,133]
***Rarb^−/−^* mice**	normal development	normal development	none	[131]
***Rarg^−/−^* mice**	↑ granulocytes in PB, BM and spleen several defects both in erythropoiesis and B lymphopoiesis	↑osteoclastogenesis ↓ trabecular bone mass	manifold defects in several tissues high post-natal mortality	[132,133,134,135,137]
***Nestin-Cre:*** ***Rarg ^Δ/Δ^* mice**	impaired BM B lymphopoiesis and thymic T lymphopoiesis normal HSCs numbers and function	NA	/	[102]
***Prrx1:Rarg^Δ/Δ^* female mice**	↑ BM granulocytes	↑ trabecular osteoblasts	enhanced BM vascularization: ↑ expression of *Vegf-a*	[127]
***Prrx1:Rarg^Δ/Δ^* male mice**	↑ pro-B and pre-B lymphocytes in BM unchanged number of splenic and mature PB B lymphocytes	↑ trabecular osteoclasts	enhanced BM vascularization: ↑ expression of *Vegf-a*	[127]

## Abbreviations

APL	acute promyelocytic leukemia
AML	acute myeloid leukemia
RBPs	retinol-binding proteins
ADHs	alcohol dehydrogenases
RAL	retinaldehyde
RA	retinoic acid
RALDHs	retinaldehyde dehydrogenases
CRABPs	cellular retinoic acid-binding proteins
RARs	retinoic acid receptors
PPARs	peroxisome proliferator–activated receptors
ATRA	all-trans retinoic acid
RXRs	retinoid X receptors
RAREs	retinoic acid response elements
HATs	histone acetyl transferases
NCoA3	nuclear receptor coactivator 3
ACTR	activator of the thyroid and RA receptor
HDACs	histone deacetylases
NCoR	nuclear receptor corepressor
ROR	retinoid-related orphan receptors
ROREs	ROR response elements
PPREs	peroxisome proliferator response elements
GluR1	glutamate receptor 1
CREB	cyclic AMP response element-binding protein
HSCs	hematopoietic stem cells
SLOs	secondary lymphoid organs
GM-CSF	granulocyte-macrophage colony-stimulating factor
Treg	regulatory T cell
FoxP3	forkhead-box-P3 transcription factor
iTreg	induced Treg
TGF-β	transforming growth factor-β
tTreg	Treg generated in the thymus
APCs	antigen-presenting cell
Ig	immunoglobulin
MAPK	mitogen-activated protein kinase
NF-kB	nuclear factor kappa-light-chain-enhancer of activated B cells
ILCs	innate lymphoid cells
PTH	parathyroid hormone
RANKL	nuclear factor kappa-β ligand
OPG	osteoprotegerin
BMP2	bone morphogenetic protein 2
BM	bone marrow
MSCs	mesenchymal stem cells
ZA	zoledronic acid
PML	promyelocytic leukemia
RARA	retinoic acid receptor-α
RARB	retinoic acid receptor-β
RARG	retinoic acid receptor-γ
NUP98	nucleoporin 98 gene
NPCs	nuclear pore complex
CPSF6	polyadenylation specific factor 6
OS	overall survival
LFS	leukemia-free survival
